# Sacral insufficiency fracture after stereotactic body radiation therapy for sacral metastasis

**DOI:** 10.1002/ccr3.1850

**Published:** 2018-10-11

**Authors:** Terufumi Kawamoto, Kei Ito, Tomohisa Furuya, Keisuke Sasai, Katsuyuki Karasawa

**Affiliations:** ^1^ Division of Radiation Oncology, Tokyo Metropolitan Cancer and Infectious Diseases Center Komagome Hospital Tokyo Japan; ^2^ Graduate School of Medicine Department of Radiation Oncology Juntendo University Tokyo Japan

**Keywords:** adverse event, bone metastasis, radiotherapy

## Abstract

Stereotactic body radiation therapy (SBRT) allows the targeting of high doses of radiation with steep dose gradients. Vertebral fracture is increasingly recognized as an adverse event after SBRT; however, no sacral fractures have been observed after SBRT. We report an extremely rare case of sacral insufficiency fracture after SBRT.

A 76‐year‐old woman presented with a myxoid chondrosarcoma at the left femur, following which a wide resection was performed. Four years later, she experienced right sciatica. A computed tomography (CT) scan revealed a tumor which was suspicious for malignancy at the right sacrum bone extending along the right sacral foramina (Figure 1A). She was diagnosed with oligo‐metastasis at the right sacral bone and received stereotactic body radiation therapy (SBRT). SBRT was administered at 24 Gy in two fractions to the right sacral bone (Figure 1B). This caused the right sciatica to disappear, and the tumor was controlled. Four months after SBRT, she experienced left buttock pain on standing up quickly and could not walk. A CT scan revealed a fracture line in the left sacral alae (Figure 1C,D). She was diagnosed with a sacral insufficiency fracture at a field edge after SBRT. The pain was relieved with rest, and the patient could walk without left buttock pain after two months.

**Figure 1:**
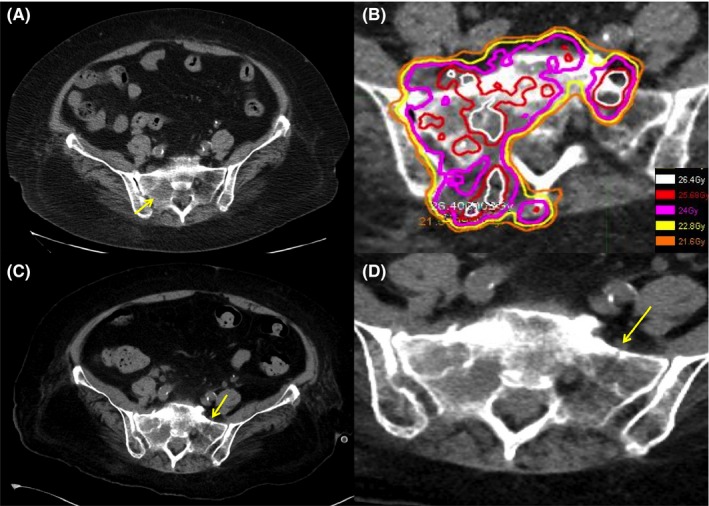
A, Axial CT scan slices. The tumor was located in the right sacral bone extending along the right sacral foramina (yellow arrow). B, Axial slices showing representative dose distribution for carcinoma. C and D, Axial slices showing the fracture line in the left sacral alae (yellow arrow)

We report an extremely rare case of sacral insufficiency fracture after SBRT. The prevalence of fracture in a review of 252 patients with 410 spinal segments was 14%; however, no sacral fractures were observed after SBRT.^1^ Previous studies have reported that age, female sex, prior vertebral compression fracture, primary hematologic malignancies, and thoracic spine tumor significantly increased the risk of fracture after SBRT.^2^ The present patient was both elderly and female, and the field edge of SBRT is a common site of sacral insufficiency fracture. These factors may cause a sacral insufficiency fracture.

In conclusion, fracture care is important for patients with risk factors after SBRT. In the case of elderly patients, maintaining safety in daily life such as using handrails is important for preventing sacral insufficiency fractures after SBRT.

## CONFLICT OF INTEREST

None declared.
